# Aversively conditioned context enhances visual size illusion via stimulus-specific neural networks

**DOI:** 10.1016/j.isci.2025.113125

**Published:** 2025-07-14

**Authors:** Jialin Zhu, Yi Yang, Lihong Chen, Wenbo Luo

**Affiliations:** 1 Institute of Psychological and Brain Sciences, Liaoning Normal University, Dalian 116029, China; 2 Key Laboratory of Brain and Cognitive Neuroscience, Dalian, Liaoning Province 116029, China

**Keywords:** Cognitive neuroscience

## Abstract

Emotion-perception interaction is a fundamental question in cognitive neuroscience, yet the underlying neural mechanisms remain poorly understood. Here, we probed this issue by combining fear conditioning paradigm with the classic Ebbinghaus illusion. We associated the surrounding context of the Ebbinghaus illusion with aversive stimuli (electric shock or intense noise) and measured behavioral and neural responses. Behaviorally, aversively conditioned contexts enhanced the perceived size illusion effect, with shock producing a stronger conditioning effect than noise. Neuroimaging revealed distinct neural pathways mediating these effects: shock conditioning strengthened occipital-to-parietal connectivity, while noise conditioning enhanced prefrontal-to-parietal connectivity. Moreover, noise relative to shock conditioning elicited greater activation in both the lateral amygdala (LA) and dorsolateral prefrontal cortex (dlPFC), and weakened LA-to-dlPFC connectivity. The findings suggest that aversive learning experiences shape human visual perception through stimulus-specific neural networks, advancing our understanding of emotion-perception interaction, and providing crucial insights for clinical interventions targeting fear-related perceptual distortions.

## Highlights

• Aversively conditioned context enhances the experienced size illusion effect
• Shock CS+ strengthens occipital-parietal connection
• Noise CS+ strengthens prefrontal-parietal connection
• Noise relative to shock CS+ weakens amygdala-prefrontal connection

## Graphical abstract

**Figure:**
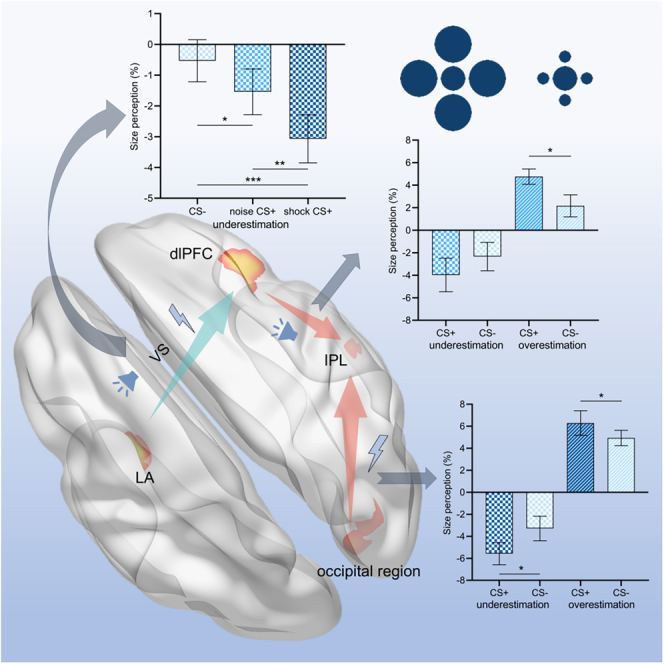

## Introduction

Fear functions as an adaptive alert system that mobilizes defensive resources in the face of danger. Converging evidence suggests that fear and threat-related signals can bias human visual perception. For instance, people are inclined to overestimate the perceived proximity,^1^ size,^2^^,^^3^ and duration^4^ of threatening objects and to underestimate the time to contact with them.^5^ Persons with spider phobia estimate the size of spiders as larger^6^ and perceive spider pictures more often as the initial percept during binocular rivalry.^7^ Moreover, it has been found that prior presentation of threatening animals (i.e., spiders and snakes) can decrease the context sensitivity of visual size perception, which is indexed by the magnitude of the Ebbinghaus illusion.^8^

The aforementioned studies elicit fear emotions by using pictures of fearful stimuli or real threatening stimuli without direct physical contact, which could produce limited threat to humans. The fear conditioning paradigm is an effective and widely used method to elicit fear in animals. It describes a learning procedure^9^ during which an initially neutral stimulus (conditioned stimulus, CS) can acquire emotional properties due to associations with an aversive event (unconditioned stimulus, US). In comparison with traditional fearful stimuli, fear conditioning can endow neutral stimuli with affective value, as demonstrated by slower heart rate and respiration amplitude, higher skin conductance, and larger pupil size,^10^^,^^11^^,^^12^^,^^13^^,^^14^^,^^15^^,^^16^ without altering their physical characteristics. Whether aversively conditioned neutral stimuli have a similar influence on early visual perception as traditional fearful stimuli remains to be explored.

The most commonly used USs are electric shock and intense noise.^17^^,^^18^^,^^19^^,^^20^ However, there has been limited research that directly compares the conditioning effects of electrotactile and auditory USs.^21^^,^^22^^,^^23^ Electrotactile fear conditioning is more related to a pain-fear-network including somatosensory, prefrontal, and parietal cortices,^24^ whereas auditory fear conditioning mainly activates a fear network consisting of the amygdala, insula, and anterior cingulate.^25^^,^^26^^,^^27^^,^^28^^,^^29^^,^^30^ To this end, we adopted electric shock and intensive noise as USs, and paired them with the surrounding inducers of the Ebbinghaus configuration, to investigate whether the conditioning effect varied as a function of the US modality (tactile and auditory). It has been shown that threatening information can automatically attract attention,^31^^,^^32^ and paying attention to the small or large contextual inducers of the Ebbinghaus configuration increases or decreases the perceived size of the central target, respectively.^33^ Thus, we predicted that when the contextual inducers were paired with the US (CS+), they would strengthen the size underestimation or overestimation of the central target relative to when the inducers were never paired with the US (CS-).

Neuroimaging research has shown that neural circuits underlying fear conditioning include a widely distributed network of structures, including amygdala, hippocampus, and prefrontal areas;^34^^,^^35^ although the functional interactions within and between these structures remain unclear. The prefrontal cortex has been suggested to regulate fear expression^36^ and extinction.^37^^,^^38^^,^^39^ For example, animal studies have shown that lesions or inactivation of the prefrontal cortex facilitates affective behaviors, whereas excitation of the prefrontal cortex suppresses similar behaviors.^40^^,^^41^^,^^42^^,^^43^ The dorsolateral prefrontal cortex (dlPFC) has tentatively been implicated in fear learning through its role in fear regulation.^44^ The amygdala is a locus for CS-US convergence and is involved in both the acquisition and extinction of fear memories.^45^^,^^46^ Lesions or inactivation of the lateral amygdala (LA) blocks the acquisition and expression of conditioned fear.^47^^,^^48^^,^^49^^,^^50^ Moreover, a high degree of synchrony between the prefrontal cortex, amygdala and hippocampus throughout different stages of fear learning has been suggested.^51^ Activity in the amygdala is regulated by inputs from the prefrontal cortex, including the dlPFC.^52^^,^^53^^,^^54^ Changes in theta coherence from the basolateral amygdala to the medial prefrontal cortex predict inter-individual variations in fear memory consolidation.^55^ Thereby, we conjectured that the modulation of aversively conditioned context on visual size perception could rely on feedback projections from cortical and/or subcortical fear-related regions (such as the prefrontal cortex and amygdala) to the parietal and occipital areas that are associated with the processing of the Ebbinghaus illusion.^56^^,^^57^^,^^58^

**Figure 1:**
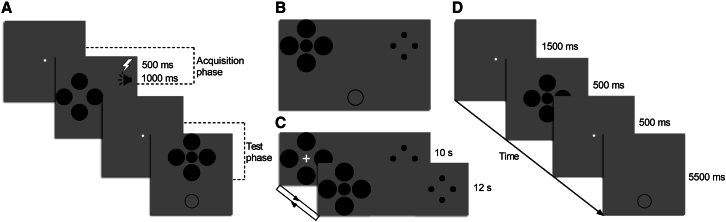
Schematic representation of experimental procedures (A) During the acquisition phase, either four large or small inducers were repeatedly paired with an electric shock (Experiment 1) or an intensive noise (Experiment 2). Participants were asked to judge the location of the inducers. During the test phase, the illusory configuration and a comparison circle were simultaneously presented. Participants had to perform the size-match task. (B) In the pilot study of Experiment 1b, four large and four small inducers were simultaneously presented in one display, and the target circle could be presented among either the four large or small inducers. Participants were required to perform the size-matching task. (C) In Experiment 1b, the surrounding inducers and a white fixation point were simultaneously presented for 10 s, and then the fixation point was replaced by the target circle for 12 s. Participants had to passively view the stimuli with no explicit task. (D) In Experiments 2b and 3b, the illusory configuration was presented for 500 ms, followed by a 500-ms fixation point, and then a comparison circle was presented for 5,500 ms, during which participants had to perform the size-matching task.

To this end, the present study investigated the neural mechanisms underlying emotion-perception interaction by combining fear conditioning with the Ebbinghaus size illusion across three studies, each comprising behavioral and neuroimaging experiments (Figure 1). Study 1 examined shock-conditioned contexts, revealing that aversive conditioning enhanced the experienced size illusion (Experiment 1a) through increased occipital-to-parietal connectivity (Experiment 1b). Study 2 explored noise-conditioned contexts, demonstrating that this conditioning strengthened the perceived size illusion (Experiment 2a) via enhanced prefrontal-to-parietal connectivity (Experiment 2b). Study 3 directly compared these conditioning types, showing that shock produced stronger perceptual effects than noise (Experiment 3a), while noise conditioning, relative to shock, elicited greater activation in both LA and dlPFC, accompanied by weakened connectivity between these regions (Experiment 3b). Together, these findings reveal that different aversive stimuli modulate visual perception through distinct neural pathways, advancing our understanding of how emotional learning shapes perceptual processes and offering potential insights for clinical interventions targeting fear-related perceptual distortions.

## Results

### Experiment 1: Contextual inducers paired with shock US

**Figure 2:**
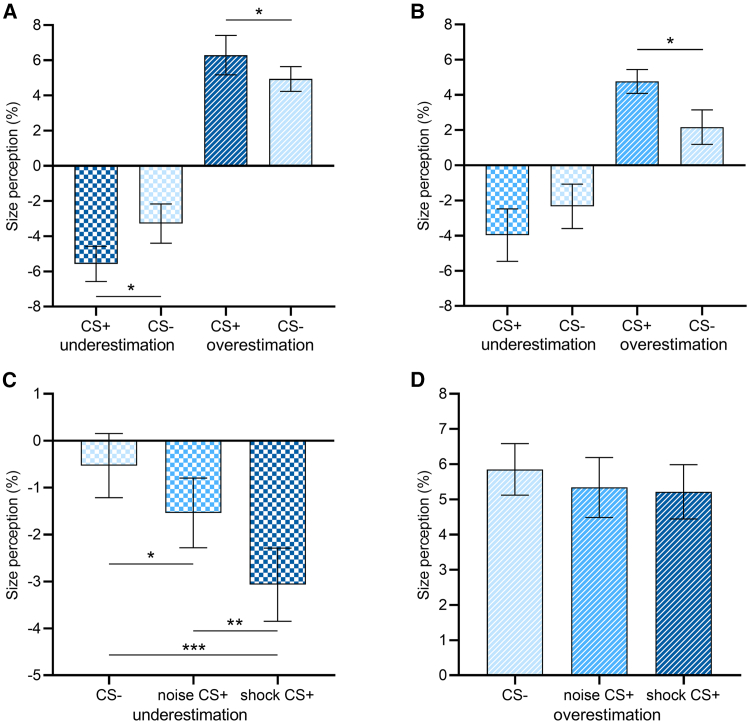
Behavioral results Perceived size (%) of the central target circle as a function of fear conditioning and inducer size in (A) Experiment 1a (*n* = 16, paired sample t test) and (B) Experiment 2a (*n* = 23, independent sample t-test). Perceived size (%) of the central target circle as a function of fear conditioning (CS-, noise CS+ and shock CS+) when surrounded by (C) large and (D) small inducers in Experiment 3a (*n* = 24, paired sample t test). Error bars represent one standard error of the mean. Asterisks indicate a significance level of ∗*p* < 0.05, ∗∗*p* < 0.01, and ∗∗∗*p* < 0.001.

In Experiment 1a, results of repeated-measures ANOVA showed that the main effect of inducer size was significant (*F*(1,15) = 52.86, *p* < 0.001, *η*_p_^2^ = 0.78), but the main effect of fear conditioning failed to reach significance (*F*(1,15) = 1.14, *p* = 0.303, *η*_p_^2^ = 0.07). Notably, there was a significant interaction between these two variables (*F*(1,15) = 6.14, *p* = 0.026, *η*_p_^2^ = 0.29). Further analysis revealed that, CS+ relative to CS- significantly increased both the underestimation (mean difference = −2.29%, *t*(15) = −2.21, *p* = 0.043, *d* = 0.56) and the overestimation (mean difference = 1.35%, *t*(15) = 2.17, *p* = 0.047, *d* = 0.54; Figure 2A) portions of the illusion effect. The behavioral results provided clear evidence that fear conditioning strengthened the experienced illusion effect.

In the pilot study of Experiment 1b, results of repeated-measures ANOVA revealed a significant main effect of target location (*F*(1,15) = 66.69, *p* < 0.001, *η*_p_^2^ = 0.82), as demonstrated that the central target was perceived as larger when presented among small inducers than large inducers. Neither the main effect of spatial layout of inducers (*F*(1,15) = 0.004, *p* = 0.950, *η*_p_^2^ < 0.001) nor the interaction of these two variables (*F*(1,15) = 0.87, *p* = 0.370, *η*_p_^2^ = 0.06) was significant (Figure S1A). The findings suggested that the current parameters could produce significant illusion effect, consistent with our previous study.^57^

### Whole brain voxel-wise results

**Figure 3:**
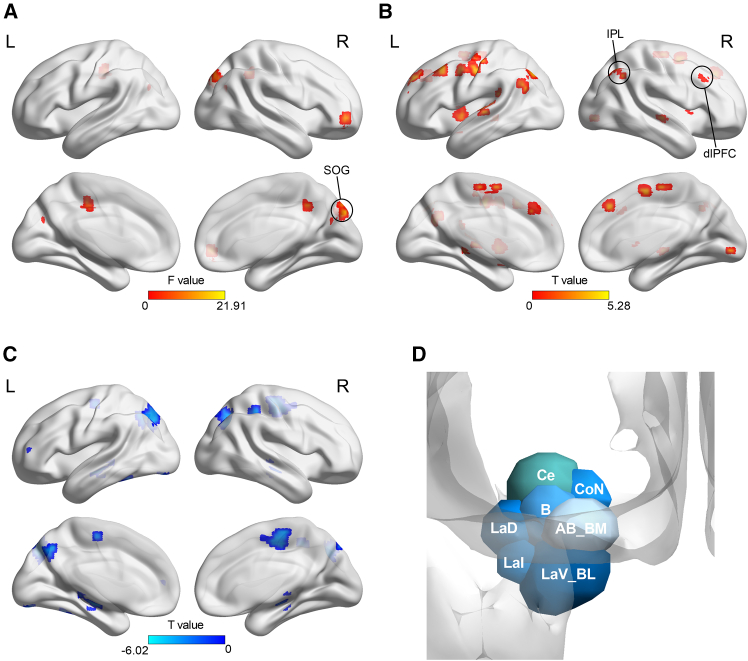
Results of group level GLM and PPI analysis in Experiment 1 (A) Brain activations in response to the interaction of target location and fear conditioning (*p* < 0.005, uncorrected, cluster size ≥10 voxels). (B and C) Brain regions showed connectivity with the occipital seed region (CS+ vs. CS-) for size (B) underestimation and (C) overestimation (*p* < 0.005, uncorrected, cluster size ≥10 voxels). (D) Schematic illustration of the subregions of left amygdala. Ce, central nucleus; CoN, cortical nuclei; B, basal; AB/BM, auxiliary basal/basomedial; LaV/BL, lateral (ventral part) containing aspects of basolateral; LaI, lateral (intermediate part); LaD, lateral (dorsal part).

Results of repeated-measures ANOVA showed that the main effect of target location (i.e., presented among small or large inducers) activated the bilateral fusiform, as well as inferior and middle occipital gyrus (Figure S2). We did not observe significant activations for the main effect of fear conditioning (*p* < 0.05, cluster-wise family-wise error [FWE] corrected). The interaction of fear conditioning and target location activated the right frontal gyrus, bilateral superior occipital gyrus (SOG, right peak MNI coordinates: 9, −84, 36; left peak MNI coordinates: −18, −75, 27), and the left middle cingulum gyrus (*p* < 0.005, uncorrected, cluster size ≥10 voxels; Figure 3A; Table S1). Further region of interest (ROI) analysis of the right SOG showed that beta values corresponded to perceived target size, with enhanced activation for stimuli eliciting larger perceived size (CS+ in overestimation condition and CS- in underestimation condition; Figure S1B).

### Psychophysiological interaction results

We performed psychophysiological interaction (PPI) analysis by comparing CS+ with CS- using the right SOG (peak MNI coordinates: 9, −84, 36; cluster size = 75 voxels) as the seed region. The seed region was functionally defined based on the activations in response to the interaction of fear conditioning (CS+ vs. CS-) and inducer size (large vs. small) at the group level (*p* < 0.005, uncorrected, cluster size ≥ 10 voxels). For size underestimation (Figure 3B), brain regions showed positive correlation with the seed region included the right inferior parietal lobule (IPL, MNI coordinates: 42, −60, 42; 23 voxels) and the right dlPFC (MNI coordinates: 51, 24, 42; 31 voxels). For size overestimation (Figure 3C), brain regions showed negative correlation with the seed region included the right frontal cortex, bilateral precuneus, and occipital regions (Table S2).

### Dynamic causal modeling (DCM) results

The four experimental conditions (underestimation CS+, underestimation CS-, overestimation CS+, and overestimation CS-), without subtracting their corresponding fixation condition, were considered as modulators of the connections among the SOG (MNI coordinates: 9, −84, 36; 75 voxels), IPL (MNI coordinates: 42, −60, 42; 23 voxels), and dlPFC (MNI coordinates: 51, 24, 42; 31 voxels). All three regions were functionally defined according to the results of the whole brain voxel-wise analysis and PPI analysis.

**Figure 4:**
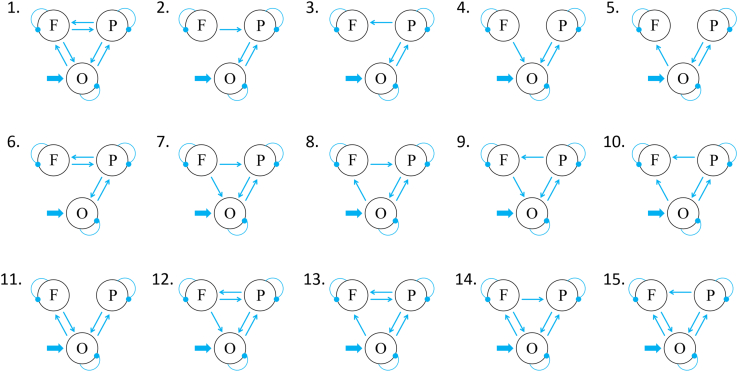
Model space of Experiments 1b and 2b The model space consisted of fifteen models which had the same driving input region, but differed in basic architecture with the bidirectional connections between the IPL and the occipital region preserved for all models.

**Figure 5:**
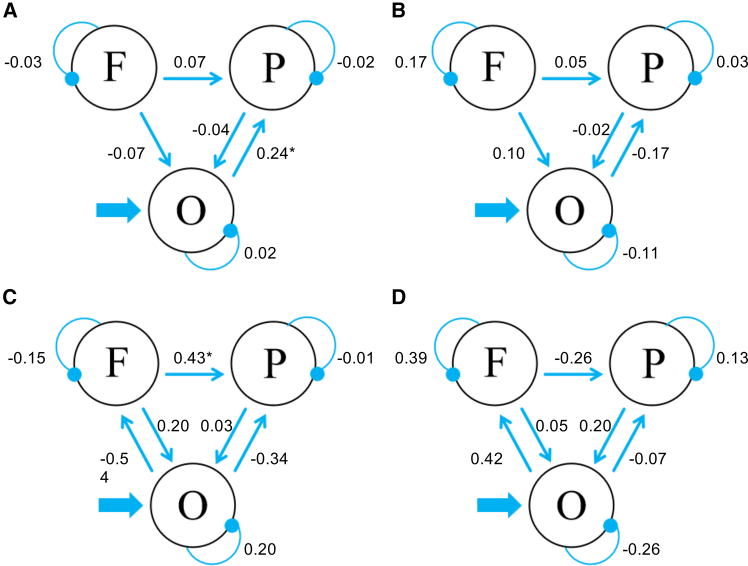
DCM results of Experiments 1 and 2 The difference of connection strength between CS+ and CS- for size underestimation and overestimation in Experiment 1b (A and B; *n* = 16, paired sample t test) and Experiment 2b (C and D; *n* = 54, independent sample t test). The values indicate the difference of connectivity strength and asterisks indicate a significance level of ∗*p* < 0.05.

Among the fifteen models (Figure 4), the winning model was model 7 (posterior probability = 0.52). For the winning model, the effect of fear conditioning (CS+ vs. CS-) on size underestimation was manifested by a significant increase of the connection strength from the SOG to IPL (M = 0.24, *t*(15) = 2.62, *p* = 0.02, *d* = 0.65; Figure 5A). We did not observe significant modulation effect of fear conditioning on size overestimation (*p*s > 0.29, uncorrected; Figure 5B).

### ROI analysis of amygdala

**Figure 6:**
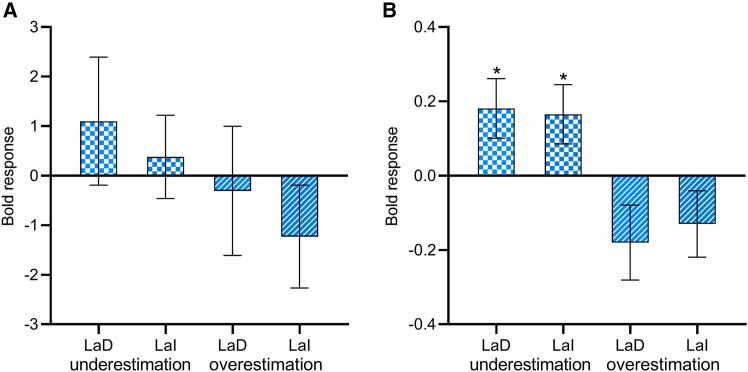
Results of amygdala ROI analysis in Experiments 1 and 2 BOLD signal differences (CS+ vs. CS-) in amygdala subregions for (A) Experiment 1b (*n* = 16, paired sample t test) and (B) Experiment 2b (*n* = 54, independent sample t tests). Error bars represent one standard error of the mean. Asterisks indicate a significance level of ∗*p* < 0.05. LaD, lateral (dorsal part); LaI, lateral (intermediate part).

We did not observe significant amygdala activation at the group level general linear model (GLM). To further explore the contribution of amygdala to the conditioning effect on visual size perception, we conducted ROI analysis of bilateral amygdala, which were defined according to automated anatomical labelling (AAL) template.^59^ We did not observe significant activations in bilateral amygdala and their subregions in response to fear conditioning for both size underestimation and overestimation (*p*s > 0.38; Figure 6A).

### Experiment 2: Contextual inducers paired with noise US

In Experiment 2a, the result showed that fear conditioning (CS+ vs. CS-) significantly strengthened size overestimation (mean difference = 2.59%, *t*(21) = 2.21, *p* = 0.038, *d* = 0.92; Figure 2B), whereas it had a negligible effect on size underestimation (mean difference = −1.63%, *t*(21) = −0.84, *p* = 0.411, *d* = 0.35).

### Whole brain voxel-wise result

**Figure 7:**
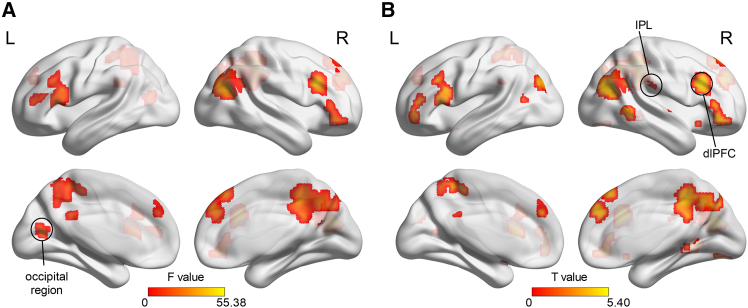
Results of group level GLM in Experiment 2 (A) Brain activations in response to the interaction of fear conditioning and inducer size (*p* < 0.05, cluster-size FWE corrected). (B) Brain activations in response to fear conditioning (CS+ vs. CS-) for size underestimation (*p* < 0.005, uncorrected, cluster size ≥10 voxels).

Results of repeated-measures ANOVA showed that the interaction of fear conditioning and inducer size activated bilateral occipital gyrus (left peak MNI coordinates: −15, −72, 12; 86 voxels), right temporal lobe, bilateral frontal gyrus, right parietal lobe, and left cingulum gyrus (*p* < 0.05, cluster-wise FWE corrected; Figure 7A; Table S3). Further analysis (independent sample t test, *p* < 0.005, uncorrected, cluster size ≥10 voxels) revealed that, for size underestimation (Figure 7B; Table S4), CS+ relative to CS- positively activated the left occipital gyrus, bilateral frontal gyrus (right peak MNI coordinates: 42, 21, 30; 145 voxels), and right parietal lobe (peak MNI coordinates: 63, −30, 21; 29 voxels).

### DCM results

Fifteen models based on the connections among the occipital region (MNI coordinates: −15, −72, 12; 86 voxels), right IPL (MNI coordinates: 63, −30, 21; 29 voxels), and right dlPFC (MNI coordinates: 42, 21, 30; 145 voxels) were set up. All the three regions were selected based on the results of whole brain voxel-wise analysis. The four experimental conditions (underestimation CS+, underestimation CS-, overestimation CS+, overestimation CS-) were considered as modulators of the connections between these sites and were incorporated into the models. The evidence for model 14 (posterior probability = 1) was higher than other models. For the winning model, the effect of fear conditioning (CS+ vs. CS-) on size underestimation was manifested by an increased connection from the dlPFC to IPL (mean difference = 0.43, *t*(52) = 2.33, *p* = 0.024, *d* = 0.63; Figure 5C). We did not observe significant conditioning effect on size overestimation (*p*s > 0.08, uncorrected; Figure 5D).

### ROI analysis of amygdala

We did not observe amygdala activation with group level GLM in Experiment 2b. To further explore the contribution of amygdala to the conditioning effect, we conducted ROI analysis of bilateral amygdala. Compared with CS-, CS+ elicited greater activity in left amygdala for size underestimation (*t* (52) = 2.27, *p* = 0.028, *d* = 0.62), and showed a similar trend for size overestimation (*t* (52) = 1.96, *p* = 0.055, *d* = 0.54). We further divided the amygdala into seven subregions (Figure 3D) according to previous literature^60^ and found that the conditioning effect on size underestimation was mainly manifested in the lateral nuclei of the left amygdala (LaD: *t*(52) = 2.17, *p* = 0.035, *d* = 0.59; LaI: *t*(52) = 2.10, *p* = 0.040, *d* = 0.57, uncorrected; Figure 6B).

### Experiment 3: Comparison of the conditioning effect between shock and noise US

Based on the DCM results of Experiments 1b and 2b, in which the conditioning effect was observed only on size underestimation rather than size overestimation, we only paired the four large inducers (size underestimation) with the USs in Experiment 3. The USs had three types: electric shock, intensive noise, and no US.

The behavioral results showed a significant main effect of US type for size underestimation (*F*(2,46) = 18.09, *p* < 0.001, *η*_p_^2^ = 0.44, Figure 2C) instead of size overestimation (*F*(2,46) = 0.810, *p* = 0.451, *η*_p_^2^ = 0.034, Figure 2D). Post-hoc analysis revealed that relative to CS-, both shock CS+ and noise CS+ significantly reduced the perceived size of the target (shock CS+: mean difference = −2.54%, *t*(23) = −7.56, *p*_*corr*_ < 0.001, *d* = 0.70; noise CS+: mean difference = −1.01%, *t*(23) = −2.28, *p*_*corr*_ = 0.032, *d* = 0.28). Notably, the conditioning-related enhancement of size underestimation was more pronounced for shock CS+ than noise CS+ (mean difference = −1.53%, *t*(23) = −3.17, *p*_*corr*_ = 0.009, *d* = 0.42). The results confirmed that fear conditioning can strengthen the experienced illusion effect, and the conditioning effect was stronger for shock than noise US. Notably, the conditioning effect specifically influenced the illusion that was paired with the US (i.e., size underestimation), while leaving the unpaired illusion (i.e., size overestimation) unaffected.

### Whole brain voxel-wise results

Under CS+ conditions (i.e., large inducers paired with shock or noise US), results of repeated-measures ANOVA showed that brain activations in response to central target relative to fixation included the bilateral occipital cortex, bilateral insula, bilateral thalamus, bilateral prefrontal cortex (right peak MNI coordinates: 51, 9, 21; 635 voxels), and bilateral inferior parietal lobule (*p* < 0.001, voxel-wise FWE corrected). We did not observe significant activations in response to neither the main effect of US type nor the interaction of them. We created a sphere of 8 mm radius at the right prefrontal cortex (dlPFC; MNI coordinates: 51, 9, 21; 86 voxels). ROI analysis of the dlPFC revealed that central target relative to fixation elicited greater activation in both CS+ condition (shock CS+: mean difference = 2.76, *t*(23) = 10.06, *p* < 0.001, *d* = 2.05; noise CS+: mean difference = 2.57, *t*(23) = 9.58, *p* < 0.001, *d* = 1.96).

### ROI analysis of amygdala

**Figure 8:**
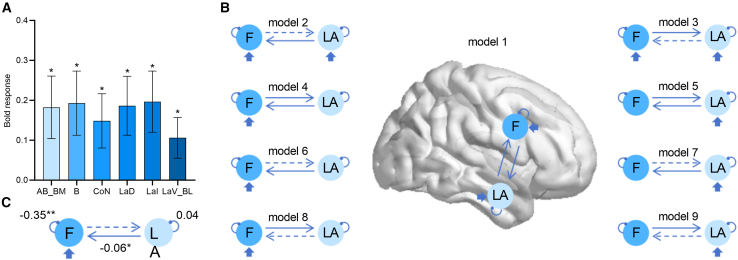
Results of Experiment 3b (A) BOLD responses to noise CS+ relative to shock CS+ in the amygdala subregions (*n* = 24, paired sample t test). (B) The model space consisted of nine models, which differed in terms of the driving input region and bidirectional connections between the dlPFC and LA. (C) The difference of connection strength between noise CS+ and shock CS+ for the winning model. Error bars represent one standard error of the mean. Asterisks indicate a significance level of ∗*p* < 0.05 and ∗∗*p* < 0.01. Ce, central nucleus; CoN, cortical nuclei; B, basal; AB/BM, auxiliary basal/basomedial; LaV/BL, lateral (ventral part) and basolateral; LaI, lateral (intermediate part); LaD, lateral (dorsal part).

We also did not observe significant amygdala activation with group level GLM. ROI analysis of bilateral amygdala with a comparison of shock CS+ versus CS-, and noise CS+ versus CS- did not reveal significant activations as well (*p*s > 0.08). When the amygdala was divided into seven subregions, noise CS+ elicited greater activation than shock CS+ (*n* = 24, paired sample t test, uncorrected; Figure 8A) in right AB_BM (*t*(23) = 2.33, *p* = 0.029, *d* = 0.48), left B (*t*(23) = 2.40, *p* = 0.025, *d* = 0.49), left CoN (*t*(23) = 2.18, *p* = 0.040, *d* = 0.45), left LaD (*t*(23) = 2.52, *p* = 0.019, *d* = 0.52), left LaI (*t*(23) = 2.27, *p* = 0.017, *d* = 0.52), and left LaV_BL (*t*(23) = 2.08, *p* = 0.049, *d* = 0.42).

### DCM result

The dlPFC and amygdala, as well as their connections have been suggested to be involved in fear conditioning.^61^^,^^62^^,^^63^^,^^64^ The left, instead of right amygdala has been found to be activated during the conscious processing of conditioned stimuli.^65^ To further explore their contributions to the conditioning effect on visual size perception, we performed DCM analysis to measure effective connections between the right dlPFC (MNI coordinates: 51, 9, 21; 84 voxels) and left LA (including left LaD and LaI; MNI coordinates: −27, −6, −27; 26 voxels). Both regions were functionally defined according to the results of whole brain voxel-wise analysis and ROI analysis. The model space consisted of nine models, which differed in terms of the driving input region and the connections between the dlPFC and LA (Figure 8B).

The evidence for model 6 (posterior probability = 1) was higher than other models. For the winning model, results showed that CS+ type (noise CS+ vs. shock CS+) was manifested by a significant decrease of inhibitory self-connection in the dlPFC (mean difference = −0.35, *t*(24) = −2.81, *p* = 0.010, *d* = 0.57) and a decreased forward connection from the LA to dlPFC (mean difference = −0.06, *t*(23) = −2.34, *p* = 0.028, *d* = 0.48; Figure 8C). We did not observe significant difference of connection strength in comparisons of neither noise CS+ vs. CS- nor shock CS+ vs. CS- (*p*s > 0.66; uncorrected).

## Discussion

By combining fear conditioning paradigm with the classic Ebbinghaus illusion, we associated the surrounding context of the Ebbinghaus illusion with aversive stimuli (i.e., electric shock or intensive noise), to investigate how fear emotion affects human visual size perception and the underlying neural mechanisms. The behavioral results showed that aversively conditioned context enhanced the experienced size illusion effect (Experiments 1a and 2a). Notably, the conditioning-related enhancement was more pronounced for shock than noise CS+ (Experiment 3a). Results of brain imaging revealed that for size underestimation, shock CS+ relative to CS- increased the connection strength from the occipital region to the parietal cortex (Experiment 1b), and noise CS+ increased the connection strength from the prefrontal to the parietal cortex (Experiment 2b). When directly comparing noise CS+ with shock CS+, noise CS+ elicited greater activation in both LA and dlPFC and weakened the connection strength from the LA to dlPFC (Experiment 3b; Table S5).

Previous studies have found that negative emotions can shape the perceived size of objects. Individuals have a tendency to perceive fearful stimuli as larger than they actually are, and this size overestimation effect extends to neutral stimuli when the individuals are experiencing a state of fear.^2^^,^^3^^,^^6^^,^^66^ The experienced size illusion effect is attenuated by prior exposure to a threatening prime.^8^ Similarly, projecting negative pictures onto the central target of the Ebbinghaus illusion significantly diminishes the illusion strength.^67^ In contrast, the current study demonstrated that associating the contextual elements of the Ebbinghaus illusion with aversive stimuli substantially enhanced the illusion strength. These results indicate that the emotional characteristics of both the central target and the contextual components of the Ebbinghaus illusion can influence the perceived size of the target, albeit in opposite directions. Stimuli that convey a sense of threat have the capacity to involuntarily capture attention.^31^^,^^68^ Therefore, we conjecture that when attention was directed to the central target, the surrounding context had less influence, resulting in a reduced size illusion effect. Conversely, attention directed to the surrounds strengthened their contextual influence, enhancing the size illusion effect. The evidence indicates that the influence of negative emotions on early visual perception is probably exerted through the deployment of spatial attention.

The commonly used USs consist of electric shocks, loud sounds, air blasts, thermal stimulation, and unpleasant odors. Only a few studies have assessed the conditioning effect using different USs. Glenn and his colleagues found that fear-potentiated startle responses were significantly higher for the shock CS+ than the scream CS+, and the shock US was rated as more aversive.^21^ Likewise, a shock US produced stronger fear conditioning as reflected in stronger acquisition and impaired extinction in contrast to an air puff to the larynx and a foul odor.^69^^,^^70^ Moreover, a shock US elicits a greater conditioning effect on the skin conductance response (SCR) during acquisition than a scream US.^71^ A meta-analysis study shows that a shock US relative to traumatic film clips produces a stronger conditioning effect on the SCR, whereas these two types of USs produce an equivalent conditioning effect on subjective rating and fear-potentiated startle.^72^ Paralleling the aforementioned findings, the current study revealed that, though the aversively conditioned context significantly strengthened the experienced size illusion effect when using both shock and noise USs, this conditioning-related enhancement was greater for the shock than the noise US. Furthermore, the present findings showed that the shock and the noise CS+ rest on distinct neural circuits, with the former associated with the occipito-parietal network and the latter linked to the fronto-parietal network. The occipito-parietal network has been consistently implicated in the processing of visual size illusions,^57^^,^^58^^,^^73^^,^^74^^,^^75^ whereas the fronto-parietal network is primarily involved in attentional control and executive functions.^76^^,^^77^^,^^78^^,^^79^ Thus, we conjecture that the stronger conditioning effect of the shock CS+ on the size illusion might be attributed to the direct modulation of the occipito-parietal network by the aversive learning experience.

The amygdala is involved in the rapid initial evaluation of and responses to threatening stimuli.^80^^,^^81^^,^^82^ Both bilateral and unilateral amygdala damage result in impaired conditioned responses, as measured by the SCR.^83^^,^^84^ Increased amygdala response to the CS+ relative to the CS- was observed for both shock and noise USs.^25^ The present study revealed similar conditioning-related enhancement of amygdala activity but only for the noise US instead of the shock US. It has been found that auditory USs such as loud screams and noise bursts are more resistant to extinction than the shock US.^23^^,^^71^ In the current study, the neuroimaging data were collected during the extinction phase, in which the USs never appeared. Therefore, the noise CS+ was more persistent than the shock CS+, as reflected in the increased amygdala activation. Notably, the conditioning effect was mainly observed in the lateral nucleus of the amygdala, which is the primary sensory input station to the amygdala, and is the key site of CS-US association during fear conditioning.^85^

The prefrontal cortex has emerged as a principal candidate for threat learning and top-down regulation of fear responses.^86^ For instance, the activity in the bilateral dlPFC can predict the awareness of the CS-US contingency^62^ and the inhibition of unwanted memories.^87^ A neutral face coupled with a shrieking female scream relative to the CS- elicits greater activation in the right dlPFC.^63^ Cognitive regulation of responses to emotional stimuli, such as negative scenes or a colored square paired with a shock, increases the blood oxygenation-level dependent (BOLD) signal in the left dlPFC, and decreases the BOLD signal in the left amygdala.^88^^,^^89^ Mirroring these findings, the current study showed that the noise CS+ relative to the CS- elicited greater activation in the right dlPFC. This was further corroborated by the DCM results from Experiment 3. In particular, compared to the shock CS+, the noise CS+ attenuated the inhibitory self-connection within the right dlPFC, indicating heightened dlPFC activation. Therefore, we speculate that the weaker contextual influence of the noise CS+ on the experienced size illusion effect might be attributed to stronger top-down regulation from the dlPFC.

Prefrontal-amygdala interactions have been implicated in the acquisition, expression, and extinction of fear conditioning. A meta-analysis of human studies reveals convergent connectivity between the amygdala and prefrontal cortex, including the right dlPFC, during emotion regulation.^90^ Inhibition of the right dlPFC by high-frequency rTMS results in increased activity within the amygdala and impaired disengagement from angry faces.^91^ Similarly, stimulation of the bilateral dlPFC via tDCS can significantly reduce the amygdala response to threat-related distractors.^92^ The directionality of prefrontal-amygdala interactions has been proposed to vary according to the different stages of fear conditioning. Specifically, during the habituation stage, dlPFC to amygdala interactions are prevalent, whereas amygdala to dlPFC interactions appear during the acquisition stage.^64^ The current study extends these findings by demonstrating that amygdala to dlPFC interactions are also engaged during the extinction stage. Moreover, the strength of this connection is more pronounced for the shock CS+ compared to the noise CS+, suggesting that the type of US also influences prefrontal-amygdala interactions.

In summary, the current study demonstrates that aversive learning experiences can influence human visual perception differently, depending on the nature of US employed. Specifically, conditioning with electric shock produces a more pronounced effect on subjective perceptual experience compared to conditioning with aversive noise. The observed difference may be attributed to the more direct influence of shock conditioning on perception-related neural networks and a comparatively diminished modulation from emotion-regulation networks. These findings illuminate the dynamic interplay between emotion, perception, and the brain, opening avenues for further research in both healthy and clinical populations.

### Limitations of the study

Although associative conditioning was confirmed at the perceptual level, physiological responses were not assessed to validate this learning process due to equipment constrains. Future investigations should incorporate autonomic measures, such as skin conductance, heart rate, and pupil diameter, to provide direct biological evidence supporting successful conditioning.

## Resource availability

### Lead contact

Requests for further information and resources should be directed to and will be fulfilled by the lead contact, Lihong Chen (lihongchen@lnnu.edu.cn).

### Materials availability

This study did not generate new unique reagents.

### Data and code availability

• All data reported in this paper will be shared by the lead contact upon request.
• This paper does not report original code.
• Any additional information required to reanalyze the data reported in this paper is available from the lead contact upon request.

## Acknowledgments

This work was supported by grants from the 10.13039/501100001809National Natural Science Foundation of China (32171047 and 32020103008), the 10.13039/501100018617Liaoning Revitalization Talents Program (XLYC2203131), the Basic Scientific Research Business Expenses of Undergraduate Universities in Liaoning Province (LS2024Q005), and the Scientific Research and Innovation Team of Liaoning Normal University.

## Author contributions

J.Z., software, investigation, formal analysis, and writing - original draft; Y.Y., software and investigation; L.C., conceptualization, methodology, supervision, project administration, and writing – review and editing; W.L., writing – review and editing.

## Declaration of interests

The authors declare no competing interests.

## STAR★Methods

### Key resources table

**Table:** 

REAGENT or RESOURCE	SOURCE	IDENTIFIER
**Deposited data**		

Behavior data	This paper	
fMRI data	This paper	

**Software and algorithms**		

MATLAB (R2022b)	MATLAB software	http://www.mathworks.com/products/matlab/
Psychophysics Toolbox-3	Brainard^93^; Pelli^94^	http://psychtoolbox.org/
JASP	University College	https://jasp-stats.org
SPM12 (V7771)	University College	https://www.fil.ion.ucl.ac.uk/spm/
RESTplus (v1.28)	University College	http://www.restfmri.net/forum/RESTplus

### Experimental model and study participant details

A total of 133 healthy participants (45 males, mean age = 22.0 years, SD = 2.5 years) took part in the study (Table S6). The sample comprised 32 participants for Experiment 1 (16 in Experiment 1a and another 16 in Experiment 1b), 77 participants for Experiment 2 (23 in Experiment 2a and 54 in Experiment 2b) and another 24 participants for Experiment 3 (the same 24 individuals completed both Experiments 3a and 3b). Nones of the participants were excluded due to excessive head movements (i.e., translation > 3 mm, or rotation > 3 degrees). Sample size was determined based on previous relevant studies.^57^^,^^95^^,^^96^ All participants were right-handed with normal or corrected-to-normal vision, and had no known neurological or psychiatric disorders. They were naive to the purpose of the study, and gave written informed consent before taking part in the experiment. The study was approved by the institutional review board of Liaoning Normal University, and complied with the Declaration of Helsinki.

### Method details

#### Stimuli and procedure

Visual stimuli were generated and presented using MATLAB (Mathworks Inc., Natick, MA, USA) and Psychophysics Toolbox-3.^93^^,^^94^ The Ebbinghaus configuration consisted of a central circle (diameter: 1.1° of visual angle) surrounded by four inducer circles that were either small (diameter = 0.6°) or large (diameter = 1.7°). The nearest distance between the inducer circles and the target circle was 0.14° of visual angle. All stimuli were black (RGB value: 0, 0, 0) and were presented on a gray (RGB value: 128, 128, 128) background. In behavioral experiments, participants were positioned 57 cm away from the computer screen (1920 × 1080 at 60 Hz) with their head fixed on a chin rest.

Electric stimulation was delivered to the participants' preferred forearm (approximately 5 cm proximal to the carpus) via a multichannel constant current stimulator (SXC-4A, Sanxia Technique Inc., China). The stimulation intensity was individually calibrated for each participant. The intensity was initially set at 1 mA and gradually increased in 0.5-mA increments until participants reported the sensation as highly uncomfortable but tolerable (Mean = 6.40 mA, SD = 2.29, range: 1.5-10 mA).

White noise (92 dB) generated by MATLAB was delivered via two stereo speakers (RI8T, EDIFIER Technique Inc., China), which were placed on both sides of the screen, at a distance of approximately 50 cm from the participants.

#### Experiment 1: Contextual inducers paired with shock US

In Experiment 1a, each participant completed two sessions (i.e., either the four small or large inducers paired with the US), with an interval of at least one week between sessions. Each session consisted of two phases. In the acquisition phase, the inducers were presented on either the left or right side of the screen (5.1° from the screen center) for 500 ms, and were simultaneously paired with a shock (reinforcement rate: 100%). Participants were required to judge the location of the inducers as accurately and quickly as possible. There were 20 trials in total, with 10 repetitions for each condition (inducer location: left or right). Prior to acquisition, participants were verbally instructed to expect a shock paired with the presentation of the inducers. In the following test phase, the illusory configuration was presented at the screen center, with a comparison circle presented below it (4.1° from the screen center). The initial diameter of the comparison circle was randomly selected from 0.83° to 1.34° in 0.057° steps on each trial. Participants had to adjust the size of the comparison circle to match that of the central target with no time limit (i.e., size-matching task; Figure 1A). Shocks were not delivered during the test phase. There were 44 trials in total, with 22 repetitions for each condition (inducer size: large or small).

The arrangement of the illusory stimuli in Experiment 1b was different from that in Experiment 1a. Thus, we first conducted a pilot behavioral experiment outside the scanner to confirm that the illusory configurations used in Experiment 1b were effective at producing the illusion effect. In particular, four large and four small inducers were simultaneously presented on the left and right sides of the screen (6.8° from the screen center), with their locations counterbalanced across trials. The central target could be presented among either the four large or four small inducers. Meanwhile, a comparison circle was presented below the illusory configuration (8.3° from the screen center). Participants had to perform the size-matching task (Figure 1B). There were 44 trials in total, with 11 repetitions for each condition (spatial layout of inducers: large inducers on the left and small inducers on the right, or vice versa; target location: among large or small inducers).

Experiment 1b also consisted of two sessions (i.e., either four large or four small inducers were paired with a shock), which were performed on separate days with an interval of at least one week. Each session was composed of two phases. The acquisition phase adopted the same stimuli and procedure as those used in Experiment 1a, and were performed outside the scanner. The test phase was conducted in the scanner and comprised four functional runs, with 110 volumes acquired per run. Each run included 10 blocks of alternating 10-s fixation and 12-s target presentation. During scanning, the context elements (i.e., four large and four small inducers) were always displayed on the screen. The positions of the large and small inducers were kept constant within each run, but were counterbalanced across runs. Within each block, the fixation point and the central target were always presented at the same location (Figure 1C), which was counterbalanced across blocks. In order to avoid task- and motor-related brain signals, participants did not perform an explicit task during scanning.^97^ Electric shocks were not delivered during scanning. The visual angles of the stimuli were linearly scaled by a factor of 1.5 to adjust their sizes to the MRI setting.

#### Experiment 2: Contextual inducers paired with noise US

We adopted a between-subjects design for both Experiments 2a and 2b, with CS (four large or small inducers) as a between-subjects factor. In particular, the CS that was paired with the US was four large inducers for half of the participants, and was four small inducers for the other half.

Experiment 2a was conducted outside the scanner, and consisted of two phases. In the acquisition phase, the surrounding inducers (either large or small) were presented on the left or right side of the screen (5.1° from the screen center) for 1000 ms and were simultaneously paired with an intensive noise (reinforcement rate: 100%). Participants were required to discriminate the location of the inducers as accurately and quickly as possible. There were a total of 330 trials, with 165 repetitions for each condition (inducer location: left or right). In the test phase, the stimuli and procedures were the same as those used in Experiment 1a. The intensive noise was not delivered during the test phase.

Experiment 2b was composed of two phases, with the acquisition and the test phases performed outside and inside the scanner, respectively. The stimuli and procedures used in the acquisition phase were the same as those in Experiment 2a. In the test phase, on each trial, a 1500-ms white fixation point was followed by a 500-ms presentation of the Ebbinghaus configuration. Then, the white fixation point reappeared for 500 ms. Finally, a comparison circle was displayed for 5500 ms, during which participants had to perform the size-matching task (Figure 1D). There were a total of 56 trials, with 28 repetitions for each condition (inducer size: large or small).

We used an event-related design and added 50% null trials among 4 functional runs, with 112 volumes acquired per run. Each run consisted of fourteen 8-sec stimulus presentation with randomly distributed 2-sec blank interval whose overall duration was 112 s. The stimulus sequence and onset times of the blank intervals were optimized using AFNI’s RSFgen program (http://afni.nimh.nih.gov), to maximize hemodynamic signal sensitivity. In our analysis, we considered only the brain responses evoked by the first 2-sec of each trial, which included the presentation of the fixation point (1500 ms) and the illusory configuration (500 ms).

#### Experiment 3: Comparison of the conditioning effect between shock and noise US

The aim of Experiment 3 utilized a within-subjects design to directly compare the effects of shock and noise conditioning on visual size perception. Based on the results of Experiments 1 and 2, only four large inducers were conditioned with an aversive US during the acquisition phase. We adopted a reinforcement rate of 75%, as a partial reinforcement contingency ratio can be used to prevent habituation to the US^63^ and to slow extinction.^54^^,^^98^^,^^99^ Both Experiments 3a and 3b comprised three sessions, which paired four large inducers (CS) with electric shock (i.e., shock session), intensive noise (i.e., noise session), or not paired them with any aversive stimuli (CS- session), respectively. The three sessions were performed on separate days with an interval of at least one week, and their sequence was counterbalanced across participants.

In Experiment 3a, each session consisted of two phases. In the acquisition phase, the shock session consisted of 32 trials, with the CS paired with a 500-ms electric shock. The noise session contained 200 trials, and adopted a 1000-ms intensive noise as US. The CS was presented for 1000 ms and 2000 ms in these two sessions, respectively, and disappeared simultaneously with the US. The CS- session comprised 32 trials with the CS presented for 1000 ms without being paired with any US. The stimuli and procedures adopted in the test phase were the same as those used in Experiment 1a.

In Experiment 3b, the acquisition and the test phase were performed outside and inside the scanner, respectively. The acquisition phase adopted the same stimuli and procedures as those in Experiment 3a. In the test phase, a 1500-ms fixation point was presented at the beginning of each trial. Then four inducers together with either the target circle or the fixation point were simultaneously presented for 500 ms at the screen center. After an interval of 500 ms, a comparison circle was presented for 5500 ms, during which participants had to perform the size-matching task. There were a total of 168 trials, with 28 repetitions for each condition (US type: shock, noise, or CS-; central stimulus: target circle or fixation).

#### MRI data acquisition

In the scanner, stimuli were back-projected via a video projector (1280 × 1024 at 60 Hz) onto a translucent screen placed inside the scanner bore. Participants viewed the stimuli through a mirror located above their eyes. Functional MRI data were collected using a 3T scanner (MR-750, GE medical systems, Milwaukee, WI) with an eight-channel phase-array coil. BOLD signals were acquired using a T2∗-weighted EPI sequence (Experiment 1b: echo time = 30 ms, repetition time = 2000 ms, field of view = 224 mm, matrix = 64 × 64, flip angle = 90 °, number of slices = 33, spatial resolution = 3.5 × 3.5 × 3.5 mm^3^; Experiments 2b and 3b: echo time = 30 ms, repetition time = 2000 ms, field of view = 192 mm, matrix = 64 × 64, flip angle = 90 °, number of slices = 43, spatial resolution = 3 × 3 × 3 mm^3^). High-resolution 3D T1-weighted anatomic images (echo time = 3 ms, repetition time = 6.9 ms, field of view = 256 mm, matrix = 256 × 256, flip angle = 8 °, number of slices = 176, spatial resolution = 1 × 1 × 1 mm^3^) were collected for each participant. To minimize head movements, straps and foam pads were used to fix the head comfortably during scanning. The first three scans of each functional run were discarded to allow for magnetic field stabilization.

#### MRI data pre-processing

Image time-series were pre-processed and analysed using RESTplus (v1.28) and SPM12 (v7771, Wellcome Trust Centre for Neuroimaging, London, UK). Images were slice-time corrected, spatially normalized into a standard stereotactic space (Montreal Neurological Institute template) and smoothed using an isotropic 6-mm Gaussian kernel. Low frequency noise was removed via high-pass filtering (cutoff 1/128 Hz), and time-series were corrected for serial autocorrelations using a first-order autoregressive [AR(1)] model. All coordinates were reported using the Montreal Neurological Institute (MNI) convention.

#### Whole brain voxel-wise analysis

We employed a mass univariate approach based on a general linear model. At the first level, models contained four event types (underestimation CS+, underestimation CS-, overestimation CS+, and overestimation CS-) during the test phase for Experiments 1b and 2b, and three event types (Shock CS+, Noise CS+, and CS-) for Experiment 3b. In addition to these event regressors, six motion parameters were included as regressors of no interest. Second-level analyses were conducted using repeated-measures ANOVA with distinct factors across experiments: Experiment 1b employed target location (presented among small or large inducers) and fear conditioning (CS+ or CS-); Experiment 2b utilized inducer size (large or small) and fear conditioning (CS+ or CS-); Experiment 3b implemented a single-factor design examining fear conditioning type (shock CS+, noise CS+, and CS-). Statistical maps underwent FWE correction with a significance threshold of *p* < 0.05.

#### Connectivity analysis

The PPI analysis^100^ was performed, using the right SOG (MNI coordinates: 9, -84, 36; cluster size = 75 voxels) as the seed region, to test for any changes in functional coupling between the seed region and each voxel in the whole brain as a function of experimental conditions (CS+ vs. CS-) in Experiment 1b. The seed region was functionally defined based on the activations in response to the interaction of fear conditioning (CS+ vs. CS-) and inducer size (large vs. small) at the group level.

We extracted the first eigenvariate of the BOLD signal from the seed region. The experimental condition (i.e., CS+ vs. CS-) was expressed as a single regressor. We estimated a general linear model with following regressors: the extracted time series in the seed region, the interaction of this time series with the experimental regressor (CS+ vs. CS-), the session and block regressors, and a constant. The first two regressors were convolved with a canonical HRF. The second-level analysis was performed by calculating one-sample t-tests on first-level contrasts of the interaction term, using a threshold of *p* < 0.005 (uncorrected) and a minimum cluster extent of 10 voxels, based on a standard gray mask.

#### DCM analysis

We carried out DCM analysis using SPM12. DCM models the hierarchical organization of the brain using self-connections within a region, as well as forward and backward connections between regions.^101^

Experiments 1b and 2b utilized shock and noise USs, respectively. In these two experiments, effective connections among the occipital gyrus, the right IPL, and the right dlPFC were analysed, based on the results of the whole brain voxel-wise analysis and PPI analysis. All three regions were functionally defined. We summarized the BOLD signal using the first eigenvariate (principal component) of voxels within the occipital region (Experiment 1b: 9, -84, 36; cluster size = 75 voxels; Experiment 2b: -15, -72, 12; cluster size = 86 voxels), the IPL (Experiment 1b: 42, -60, 42; cluster size = 23 voxels; Experiment 2b: 63, -30, 21; cluster size = 29 voxels), and the dlPFC (Experiment 1b: 51, 24, 42; cluster size = 31 voxels; Experiment 2b: 42, 21, 30; cluster size = 145 voxels) for each participant. It should be noted that, we adopted the right and left occipital region as the ROI in Experiments 1b and 2b, respectively, as previous studies have found that both regions contributes to the processing of visual size illusions.^102^^,^^103^

We extracted time-series from the three ROIs and specified fifteen models for each participant, which had the same driving input region, but differed in their basic architecture. Bidirectional connections between the occipital region and the parietal region, which are associated with the processing of the Ebbinghaus illusion,^57^^,^^58^ were preserved in all fifteen models. All four experimental conditions (underestimation CS+, underestimation CS-, overestimation CS+, and overestimation CS-) were included as driving inputs that entered the occipital region. Effective connectivity analysis between the three regions was divided into (i) making different connections between the dlPFC and the IPL, as well as between the dlPFC and the occipital region for each model, and (ii) establishing a model by analyzing the connections among the regions via parametric analysis. We used Bayesian Model Reduction to evaluate the evidence for the fifteen models, and reported the posterior estimates under each model.

Experiment 3b employed both shock and noise USs in separate sessions. Effective connections between the right dlPFC (MNI coordinates: 51, 9, 21; cluster size = 84 voxels) and the left LA (MNI coordinates: -27, -6, -27; cluster size = 26 voxels) were calculated, on the basis of the results of whole brain voxel-wise analysis and ROI analysis. The model space consisted of nine models, which differed in terms of the driving input region and the connections between the dlPFC and the LA (Figure 8B). Three experimental conditions (noise CS+, shock CS+, CS-) were considered as modulators of the connections between the two sites, and were incorporated into the models.

### Quantification and statistical analysis

Significance testing of behavioral data, ROI analysis and DCM analysis were performed using the JASP. Significance testing of fMRI data was performed using the SPM (key resources table). The overestimation (or underestimation) portion of the illusion effect was measured as the perceived size of the central target surrounded by small (or large) inducers relative to its physical size (%). In Experiment 1, we performed a 2 × 2 repeated-measures ANOVA with fear conditioning (CS+ and CS-) and inducer size (large and small) as within-subjects factors. In Experiment 2, a repeated-measures ANOVA was conducted using fear conditioning (CS+ and CS-) as a between-subjects factor, and inducer size (large and small) as a within-subjects factor. In Experiment 3, a repeated-measures ANOVA was performed with fear conditioning (noise CS+, shock CS+, and CS-) as a within-subjects factor. Paired or independent sample t-tests were performed for further analysis. Partial eta-squared and Cohen’s d were calculated to assess effect sizes. To control for multiple comparisons, we applied Holm correction for behavioral analysis and FWE correction for neuroimaging analysis. The significance level was set to ɑ = 0.05. Unless otherwise specified, 95% confidence interval (SEM∗1.96) was used to quantify precision. The specific statistical tests, significance levels, and precision measurements are reported in the text and/or figure captions.

## References

[1] Cole S., Balcetis E., Dunning D. (2013). Affective signals of threat increase perceived proximity. Psychol. Sci..

[2] Chen L., Yuan X., Xu Q., Wang Y., Jiang Y. (2016). Subliminal impending collision increases perceived object size and enhances pupillary light reflex. Front. Psychol..

[3] Vasey M.W., Vilensky M.R., Heath J.H., Harbaugh C.N., Buffington A.G., Fazio R.H. (2012). It was as big as my head, I swear!: Biased spider size estimation in spider phobia. J. Anxiety Disord..

[4] Tipples J. (2011). When time stands still: Fear-specific modulation of temporal bias due to threat. Emotion.

[5] Vagnoni E., Lourenco S.F., Longo M.R. (2012). Threat modulates perception of looming visual stimuli. Curr. Biol..

[6] Leibovich T., Cohen N., Henik A. (2016). Itsy bitsy spider? Valence and self-relevance predict size estimation. Biol. Psychol..

[7] Müller U.W.D., Gerdes A.B.M., Alpers G.W. (2022). You see what you avoid: Fear of spiders and avoidance are associated with predominance of spiders in binocular rivalry. J. Anxiety Disord..

[8] Hu X., Feng B., Chen L., Luo W. (2023). Threat shapes visual context sensitivity selectively through low-spatial-frequency channels. Cognition.

[9] LeDoux J.E. (2014). Coming to terms with fear. Proc. Natl. Acad. Sci. USA.

[10] Castaneda J.O., Segerstrom S.C. (2004). Effect of stimulus type and worry on physiological response to fear. J. Anxiety Disord..

[11] Castegnetti G., Tzovara A., Staib M., Paulus P.C., Hofer N., Bach D.R. (2016). Modeling fear-conditioned bradycardia in humans. Psychophysiology.

[12] Castegnetti G., Tzovara A., Staib M., Gerster S., Bach D.R. (2017). Assessing fear learning via conditioned respiratory amplitude responses. Psychophysiology.

[13] Greenberg T., Carlson J.M., Cha J., Hajcak G., Mujica-Parodi L.R. (2013). Neural reactivity tracks fear generalization gradients. Biol. Psychol..

[14] Hermans E.J., Kanen J.W., Tambini A., Fernández G., Davachi L., Phelps E.A. (2017). Persistence of amygdala-hippocampal connectivity and multi-voxel correlation structures during awake rest after fear learning predicts long-term expression of fear. Cereb. Cortex.

[15] Moratti S., Keil A., Miller G.A. (2006). Fear but not awareness predicts enhanced sensory processing in fear conditioning. Psychophysiology.

[16] Sperl M.F.J., Wroblewski A., Mueller M., Straube B., Mueller E.M. (2021). Learning dynamics of electrophysiological brain signals during human fear conditioning. Neuroimage.

[17] Duits P., Cath D.C., Lissek S., Hox J.J., Hamm A.O., Engelhard I.M., van den Hout M.A., Baas J.M.P. (2015). Updated meta-analysis of classical fear conditioning in the anxiety disorders. Depress. Anxiety.

[18] Fullana M.A., Harrison B.J., Soriano-Mas C., Vervliet B., Cardoner N., Àvila-Parcet A., Radua J. (2016). Neural signatures of human fear conditioning: An updated and extended meta-analysis of fMRI studies. Mol. Psychiatry.

[19] Hofmann W., De Houwer J., Perugini M., Baeyens F., Crombez G. (2010). Evaluative conditioning in humans: A meta-analysis. Psychol. Bull..

[20] Lissek S., Powers A.S., McClure E.B., Phelps E.A., Woldehawariat G., Grillon C., Pine D.S. (2005). Classical fear conditioning in the anxiety disorders: A meta-analysis. Behav. Res. Ther..

[21] Glenn C.R., Lieberman L., Hajcak G. (2012). Comparing electric shock and a fearful screaming face as unconditioned stimuli for fear learning. Int. J. Psychophysiol..

[22] Luck C.C., Patterson R.R., Lipp O.V. (2023). The influence of cross unconditional stimulus reinstatement on electrodermal responding and conditional stimulus valence in differential fear conditioning. Psychophysiology.

[23] Sperl M.F.J., Panitz C., Hermann C., Mueller E.M. (2016). A pragmatic comparison of noise burst and electric shock unconditioned stimuli for fear conditioning research with many trials: Fear conditioning with noise burst vs. shock as US. Psychophysiology.

[24] Sehlmeyer C., Schöning S., Zwitserlood P., Pfleiderer B., Kircher T., Arolt V., Konrad C. (2009). Human fear conditioning and extinction in neuroimaging: A systematic review. PLoS One.

[25] Büchel C., Morris J., Dolan R.J., Friston K.J. (1998). Brain systems mediating aversive conditioning: An event-related fMRI study. Neuron.

[26] Büchel C., Dolan R.J., Armony J.L., Friston K.J. (1999). Amygdala-hippocampal involvement in human aversive trace conditioning revealed through event-related functional magnetic resonance imaging. J. Neurosci..

[27] Dunsmoor J.E., Bandettini P.A., Knight D.C. (2007). Impact of continuous versus intermittent CS-UCS pairing on human brain activation during pavlovian fear conditioning. Behav. Neurosci..

[28] Knight D.C., Nguyen H.T., Bandettini P.A. (2005). The role of the human amygdala in the production of conditioned fear responses. Neuroimage.

[29] Morris J.S., Friston K.J., Dolan R.J. (1997). Neural responses to salient visual stimuli. Proc. Biol. Sci..

[30] Morris J.S., Dolan R.J. (2004). Dissociable amygdala and orbitofrontal responses during reversal fear conditioning. Neuroimage.

[31] Lin J.Y., Franconeri S., Enns J.T. (2008). Objects on a collision path with the observer demand attention. Psychol. Sci..

[32] Koster E.H.W., Crombez G., Van Damme S., Verschuere B., De Houwer J. (2004). Does imminent threat capture and hold attention?. Emotion.

[33] Shulman G.L. (1992). Attentional modulation of size contrast. Q. J. Exp. Psychol..

[34] Etkin A., Wager T.D. (2007). Functional neuroimaging of anxiety: A meta-analysis of emotional processing in PTSD, social anxiety disorder, and specific phobia. Am. J. Psychiatry.

[35] Mechias M.-L., Etkin A., Kalisch R. (2010). A meta-analysis of instructed fear studies: Implications for conscious appraisal of threat. Neuroimage.

[36] Corcoran K.A., Quirk G.J. (2007). Activity in prelimbic cortex is necessary for the expression of learned, but not innate, fears. J. Neurosci..

[37] Adhikari A., Lerner T.N., Finkelstein J., Pak S., Jennings J.H., Davidson T.J., Ferenczi E., Gunaydin L.A., Mirzabekov J.J., Ye L. (2015). Basomedial amygdala mediates top-down control of anxiety and fear. Nature.

[38] Marek R., Sun Y., Sah P. (2019). Neural circuits for a top-down control of fear and extinction. Psychopharmacology.

[39] Milad M.R., Quirk G.J. (2012). Fear extinction as a model for translational neuroscience: Ten years of progress. Annu. Rev. Psychol..

[40] al Maskati H.A., Zbrozyna A.W. (1989). Stimulation in prefrontal cortex area inhibits cardiovascular and motor components of the defence reaction in rats. J. Auton. Nerv. Syst..

[41] Jaskiw G.E., Weinberger D.R. (1990). Ibotenic acid lesions of the medial prefrontal cortex potentiate FG-7142-induced attenuation of exploratory activity in the rat. Pharmacol. Biochem. Behav..

[42] Sacchetti B., Baldi E., Lorenzini C.A., Bucherelli C. (2002). Differential contribution of some cortical sites to the formation of memory traces supporting fear conditioning. Exp. Brain Res..

[43] Vouimba R.M., Garcia R., Baudry M., Thompson R.F. (2000). Potentiation of conditioned freezing following dorsomedial prefrontal cortex lesions does not interfere with fear reduction in mice. Behav. Neurosci..

[44] Lau J.Y., Britton J.C., Nelson E.E., Angold A., Ernst M., Goldwin M., Grillon C., Leibenluft E., Lissek S., Norcross M. (2011). Distinct neural signatures of threat learning in adolescents and adults. Proc. Natl. Acad. Sci. USA.

[45] Fanselow M.S., LeDoux J.E. (1999). Why we think plasticity underlying pavlovian fear conditioning occurs in the basolateral amygdala. Neuron.

[46] LeDoux J.E. (2000). Emotion circuits in the brain. Annu. Rev. Neurosci..

[47] Bauer E.P., Schafe G.E., LeDoux J.E. (2002). NMDA receptors and L-type voltage-gated calcium channels contribute to long-term potentiation and different components of fear memory formation in the lateral amygdala. J. Neurosci..

[48] Goosens K.A., Maren S. (2001). Contextual and auditory fear conditioning are mediated by the lateral, basal, and central amygdaloid nuclei in rats. Learn. Mem..

[49] Nader K., Majidishad P., Amorapanth P., LeDoux J.E. (2001). Damage to the lateral and central, but not other, amygdaloid nuclei prevents the acquisition of auditory fear conditioning. Learn. Mem..

[50] Wilensky A.E., Schafe G.E., LeDoux J.E. (1999). Functional inactivation of the amygdala before but not after auditory fear conditioning prevents memory formation. J. Neurosci..

[51] Giustino T.F., Maren S. (2015). The role of the medial prefrontal cortex in the conditioning and extinction of fear. Front. Behav. Neurosci..

[52] Gottfried J.A., Dolan R.J. (2004). Human orbitofrontal cortex mediates extinction learning while accessing conditioned representations of value. Nat. Neurosci..

[53] Ochsner K.N., Gross J.J. (2005). The cognitive control of emotion. Trends Cogn. Sci..

[54] Phelps E.A., Delgado M.R., Nearing K.I., LeDoux J.E. (2004). Extinction learning in humans: Role of the amygdala and vmPFC. Neuron.

[55] Popa D., Duvarci S., Popescu A.T., Léna C., Paré D. (2010). Coherent amygdalocortical theta promotes fear memory consolidation during paradoxical sleep. Proc. Natl. Acad. Sci. USA.

[56] Chen L., Zhu S., Feng B., Zhang X., Jiang Y. (2022). Altered effective connectivity between lateral occipital cortex and superior parietal lobule contributes to manipulability-related modulation of the Ebbinghaus illusion. Cortex.

[57] Chen L., Wu B., Yu H., Sperandio I. (2024). Network dynamics underlying alterations in apparent object size. Brain Commun..

[58] Wu B., Feng B., Han X., Chen L., Luo W. (2023). Intrinsic excitability of human right parietal cortex shapes the experienced visual size illusions. Cereb. Cortex.

[59] Rolls E.T., Huang C.-C., Lin C.-P., Feng J., Joliot M. (2020). Automated anatomical labelling atlas 3. Neuroimage.

[60] Klein-Flügge M.C., Jensen D.E.A., Takagi Y., Priestley L., Verhagen L., Smith S.M., Rushworth M.F.S. (2022). Relationship between nuclei-specific amygdala connectivity and mental health dimensions in humans. Nat. Hum. Behav..

[61] Büchel C., Holmes A.P., Rees G., Friston K.J. (1998). Characterizing stimulus–response functions using nonlinear regressors in parametric fMRI experiments. Neuroimage.

[62] Carter R.M., O’Doherty J.P., Seymour B., Koch C., Dolan R.J. (2006). Contingency awareness in human aversive conditioning involves the middle frontal gyrus. Neuroimage.

[63] Chauret M., Suffren S., Pine D.S., Nassim M., Saint-Amour D., Maheu F.S. (2019). Fear conditioning and extinction in anxious youth, offspring at-risk for anxiety and healthy comparisons: An fMRI study. Biol. Psychol..

[64] Liu C.C., Crone N.E., Franaszczuk P.J., Cheng D.T., Schretlen D.S., Lenz F.A. (2011). Fear conditioning is associated with dynamic directed functional interactions between and within the human amygdala, hippocampus, and frontal lobe. Neuroscience.

[65] Morris J.S., Ohman A., Dolan R.J. (1998). Conscious and unconscious emotional learning in the human amygdala. Nature.

[66] Shiban Y., Fruth M.B., Pauli P., Kinateder M., Reichenberger J., Mühlberger A. (2016). Treatment effect on biases in size estimation in spider phobia. Biol. Psychol..

[67] Van Ulzen N.R., Semin G.R., Oudejans R.R.D., Beek P.J. (2008). Affective stimulus properties influence size perception and the Ebbinghaus illusion. Psychol. Res..

[68] Koster E.H.W., Crombez G., Verschuere B., De Houwer J. (2004). Selective attention to threat in the dot probe paradigm: Differentiating vigilance and difficulty to disengage. Behav. Res. Ther..

[69] Busch C.J., Evans I.M. (1977). The effectiveness of electric shock and foul odor as unconditioned stimuli in classical aversive conditioning. Behav. Res. Ther..

[70] Murray H.G., Carruthers B.C. (1974). Human eyelid conditioning with airpuff vs infraorbital shock as the UCS. Can. J. Psychol..

[71] Ney L.J., Nichols D.S., Lipp O.V. (2023). Fear conditioning depends on the nature of the unconditional stimulus and may be related to hair levels of endocannabinoids. Psychophysiology.

[72] Ney L.J., O’Donohue M.P., Lowe B.G., Lipp O.V. (2022). Angry and fearful compared to happy or neutral faces as conditional stimuli in human fear conditioning: A systematic review and meta-analysis. Neurosci. Biobehav. Rev..

[73] Fang F., Boyaci H., Kersten D., Murray S.O. (2008). Attention-dependent representation of a size illusion in human V1. Curr. Biol..

[74] Murray S.O., Boyaci H., Kersten D. (2006). The representation of perceived angular size in human primary visual cortex. Nat. Neurosci..

[75] Song C., Sandberg K., Andersen L.M., Blicher J.U., Rees G. (2017). Human occipital and parietal GABA selectively influence visual perception of orientation and size. J. Neurosci..

[76] Kastner S., Pinsk M.A., De Weerd P., Desimone R., Ungerleider L.G. (1999). Increased activity in human visual cortex during directed attention in the absence of visual stimulation. Neuron.

[77] Moratti S., Saugar C., Strange B.A. (2011). Prefrontal-occipitoparietal coupling underlies late latency human neuronal responses to emotion. J. Neurosci..

[78] Rodríguez-Nieto G., Seer C., Sidlauskaite J., Vleugels L., Van Roy A., Hardwick R., Swinnen S. (2022). Inhibition, shifting and updating: Inter and intra-domain commonalities and differences from an executive functions activation likelihood estimation meta-analysis. Neuroimage.

[79] Silver M.A., Kastner S. (2009). Topographic maps in human frontal and parietal cortex. Trends Cogn. Sci..

[80] Lang P.J., Bradley M.M. (2010). Emotion and the motivational brain. Biol. Psychol..

[81] Popov T., Steffen A., Weisz N., Miller G.A., Rockstroh B. (2012). Cross-frequency dynamics of neuromagnetic oscillatory activity: Two mechanisms of emotion regulation. Psychophysiology.

[82] Winecoff A., Labar K.S., Madden D.J., Cabeza R., Huettel S.A. (2011). Cognitive and neural contributors to emotion regulation in aging. Soc. Cogn. Affect. Neurosci..

[83] Bechara A., Tranel D., Damasio H., Adolphs R., Rockland C., Damasio A.R. (1995). Double dissociation of conditioning and declarative knowledge relative to the amygdala and hippocampus in humans. Science.

[84] LaBar K.S., LeDoux J.E., Spencer D.D., Phelps E.A. (1995). Impaired fear conditioning following unilateral temporal lobectomy in humans. J. Neurosci..

[85] Herry C., Johansen J.P. (2014). Encoding of fear learning and memory in distributed neuronal circuits. Nat. Neurosci..

[86] Alexandra Kredlow M., Fenster R.J., Laurent E.S., Ressler K.J., Phelps E.A. (2022). Prefrontal cortex, amygdala, and threat processing: Implications for PTSD. Neuropsychopharmacology.

[87] Anderson M.C., Ochsner K.N., Kuhl B., Cooper J., Robertson E., Gabrieli S.W., Glover G.H., Gabrieli J.D.E. (2004). Neural systems underlying the suppression of unwanted memories. Science.

[88] Delgado M.R., Nearing K.I., Ledoux J.E., Phelps E.A. (2008). Neural circuitry underlying the regulation of conditioned fear and its relation to extinction. Neuron.

[89] Ochsner K.N., Bunge S.A., Gross J.J., Gabrieli J.D.E. (2002). Rethinking feelings: An fMRI study of the cognitive regulation of emotion. J. Cogn. Neurosci..

[90] Berboth S., Morawetz C. (2021). Amygdala-prefrontal connectivity during emotion regulation: A meta-analysis of psychophysiological interactions. Neuropsychologia.

[91] De Raedt R., Leyman L., Baeken C., Van Schuerbeek P., Luypaert R., Vanderhasselt M.A., Dannlowski U. (2010). Neurocognitive effects of HF-rTMS over the dorsolateral prefrontal cortex on the attentional processing of emotional information in healthy women: An event-related fMRI study. Biol. Psychol..

[92] Ironside M., Browning M., Ansari T.L., Harvey C.J., Sekyi-Djan M.N., Bishop S.J., Harmer C.J., O'Shea J. (2019). Effect of prefrontal cortex stimulation on regulation of amygdala response to threat in individuals with trait anxiety: A randomized clinical trial. JAMA Psychiatry.

[93] Brainard D.H. (1997). The Psychophysics Toolbox. Spat. Vis..

[94] Pelli D.G. (1997). The videotoolbox software for visual psychophysics: Transforming numbers into movies. Spat. Vis..

[95] Chen L., Qiao C., Wang Y., Jiang Y. (2018). Subconscious processing reveals dissociable contextual modulations of visual size perception. Cognition.

[96] Wang A., Chen L., Jiang Y. (2021). Anodal occipital transcranial direct current stimulation enhances perceived visual size illusions. J. Cogn. Neurosci..

[97] Weidner R., Boers F., Mathiak K., Dammers J., Fink G.R. (2010). The temporal dynamics of the Müller-Lyer illusion. Cereb. Cortex.

[98] Chan C.K.J., Harris J.A. (2019). The partial reinforcement extinction effect: The proportion of trials reinforced during conditioning predicts the number of trials to extinction. J. Exp. Psychol. Anim. Learn. Cogn..

[99] Haselgrove M., Aydin A., Pearce J.M. (2004). A partial reinforcement extinction effect despite equal rates of reinforcement during pavlovian conditioning. J. Exp. Psychol. Anim. Behav. Process..

[100] Friston K.J., Buechel C., Fink G.R., Morris J., Rolls E., Dolan R.J. (1997). Psychophysiological and modulatory interactions in neuroimaging. Neuroimage.

[101] Lumaca M., Dietz M.J., Hansen N.C., Quiroga-Martinez D.R., Vuust P. (2021). Perceptual learning of tone patterns changes the effective connectivity between Heschl’s gyrus and planum temporale. Hum. Brain Mapp..

[102] Von Gal A., Boccia M., Nori R., Verde P., Giannini A.M., Piccardi L. (2023). Neural networks underlying visual illusions: An activation likelihood estimation meta-analysis. Neuroimage.

[103] Zeng H., Fink G.R., Weidner R. (2020). Visual size processing in early visual cortex follows lateral occipital cortex involvement. J. Neurosci..

