# Supplementary material for: Aversively conditioned context enhances visual size illusion via stimulus-specific neural networks
Source: iScience. 2025 Jul 14;28(8):113125. doi: 10.1016/j.isci.2025.113125 (PMC12329298; doi:10.1016/j.isci.2025.113125)
Supplement: Document S1. Figures S1 and S2 and Tables S1–S6 [file mmc1.pdf]

iScience, Volume *28*

Supplemental information

Aversively conditioned context enhances visual size illusion via stimulus-specific neural networks

Jialin Zhu, Yi Yang, Lihong Chen, and Wenbo Luo

Supplementary Figures

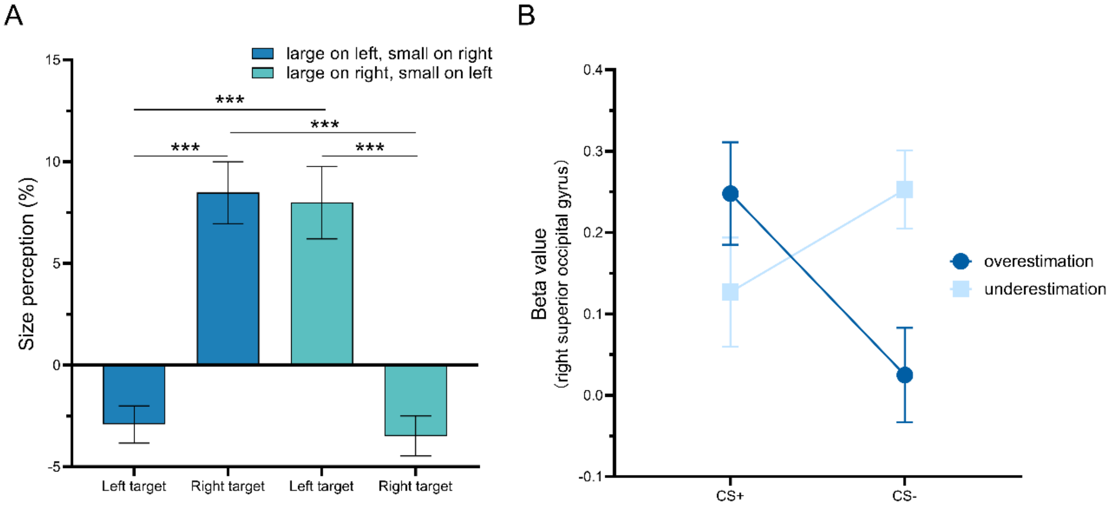

Figure S1. Results of (A) pilot study and (B) ROI analysis of the right superior occipital gyrus in Experiment 1b. Error bars represent one standard error of the mean. Asterisks indicate a significance level of ***$p < 0.001$.

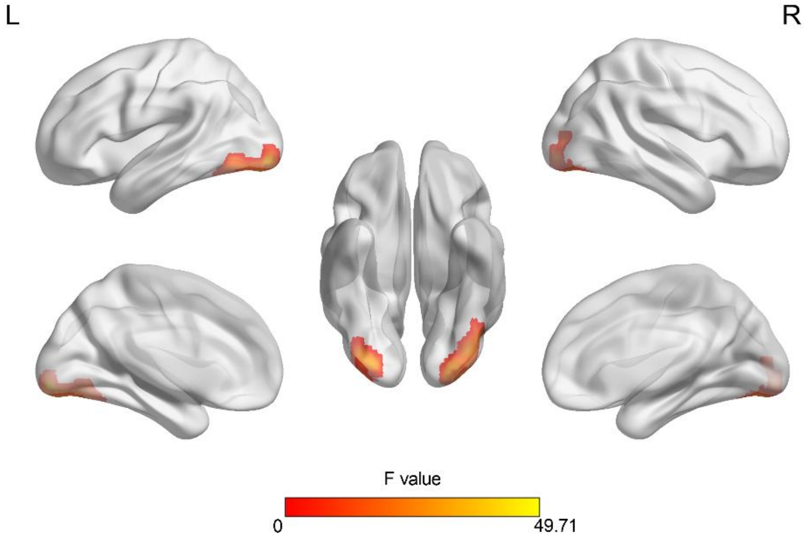

Figure S2. Brain activations in response to inducer size in Experiment 1b ($p < 0.005$, uncorrected, with cluster size ≥ 10 voxels).

Supplementary tables

Table S1. Brain regions showing significant interaction of target location and fear conditioning in Experiment 1b (p < 0.005, uncorrected, cluster size ≥ 10 voxels).

Region	Hemi.	Peak MNI coordinates	Cluster size
Frontal gyrus	R	36, 45, -6	33
Superior occipital lobe	R	9, -84, 36	75
Superior occipital lobe	L	-18, -75, 27	11
Middle cingulum gyrus	L	9, 27, -42	16
Precuneus	R	12, -48, 42	10

Table S2. Results of PPI analysis (CS+ vs. CS-) in Experiment 1b (p < 0.005, uncorrected, cluster size ≥ 10 voxels).

Condition	Region	Hemi.	Peak MNI coordinates	Cluster size
Underestimation	Putamen	L	-24, 6, -9	41
	Putamen	R	33, 6, 0	62
	Insula	L	-36, -6, -6	23
	Caudate	L	-9, 15, 6	32
	Frontal gyrus	L	-18, 48, 39	23
	Parietal lobule	L	-45, -60, 33	59
	Middle frontal gyrus	R	51, 24, 42	31
	Inferior parietal lobule	R	42, -60, 42	23
	Middle frontal gyrus	L	-45, 24, 45	41
Overestimation	Hippocampus	L	-21, -18, -15	33
	Thalamus	R	15, -24, 6	38
	Middle occipital gyrus	L	-27, -75, 39	27
	Precuneus	R	24, -66, 42	67

	Frontal gyrus	R	12, -12, 51	76

Table S3. Brain regions exhibiting significant interaction of inducer size and fear conditioning in Experiment 2b ($p < 0.05$, cluster-wise FWE corrected).

**Region**	**Hemi.**	**Peak MNI coordinates**	**Cluster size**
Frontal gyrus	R	48, 39, -9	86
Occipital lobe	L	-15, -72, 12	86
Frontal gyrus	L	-45, 15, 12	227
Middle frontal gyrus	R	42, 21, 30	157
Medial frontal gyrus	R	3, 48, 36	180
Parietal lobe	R	9, -39, 42	574

Table S4. Brain regions in response to fear conditioning (CS+ vs. CS-) in Experiment 2b ($p < 0.005$, uncorrected, cluster size ≥ 10 voxels).

**Condition**	**Region**	**Hemi.**	**Peak MNI coordinates**	**Cluster size**
Underestimation	Inferior frontal gyrus	R	42, 42, -12	73
	Temporal lobe	R	60, -54, 0	51
	Inferior frontal gyrus	L	-45,15,12	144
	Middle temporal gyrus	R	42, -72, 21	195
	Inferior parietal lobule	R	63, -30,21	29
	Middle frontal gyrus	R	42,21,30	145
	Media frontal gyrus	R	3,48,36	102
	Parietal lobe	R	9,-39,42	383
	Superior frontal gyrus	R	6,36,51	47
	Occipital gyrus	R	21, -81, -9	26

Table S5. Summary of principal findings across all experiments.

Experiment	Key findings
1a	Shock-conditioned context enhanced the experienced size illusion effect.
Pilot 1b	The layout of illusory configuration was effective at eliciting size illusion effect at the behavioral level.
1b	Enhanced size illusion following shock conditioning was mediated by increased occipital-to-parietal connectivity.
2a	Noise-conditioned context strengthened the overestimation portion of the size illusion effect.
2b	Noise conditioning enhanced the size illusion through strengthened prefrontal-to-parietal connectivity.
3a	The enhancement of perceived size illusion following conditioning was more pronounced for shock than noise US.
3b	Noise relative to shock conditioning elicited greater activation in both lateral amygdala (LA) and dorsolateral prefrontal cortex (dlPFC), and weakened LA-to-dlPFC connectivity.

Table S6. Methodological details for all experiments.

Experiment	Phase	Scanner	Task	Trial number	Sample size
1a (behavior)	acquisition	outside	location discrimination	20	16
	test		size-matching	44	
Pilot 1b (behavior)	-	outside	size-matching	44	16
1b (fMRI)	acquisition	outside	location discrimination	20	
	test	inside	passive viewing	10	
2a (behavior)	acquisition	outside	location discrimination	330	23
	test		size-matching	44	
2b (fMRI)	acquisition	outside	location discrimination	330	54
	test	inside	size-matching	56	
3a (behavior)	acquisition	outside	location discrimination	shock: 32<br>noise: 200<br>no US: 32	24
	test		size-matching	44	
3b (fMRI)	acquisition	outside	location discrimination	shock: 32<br>noise: 200<br>no US: 32	
	test	inside	size-matching	56	
